# Reduced Fibroblast Activation on Electrospun Polycaprolactone Scaffolds

**DOI:** 10.3390/bioengineering10030348

**Published:** 2023-03-11

**Authors:** Joe P. Woodley, Daniel W. Lambert, Ilida Ortega Asencio

**Affiliations:** The School of Clinical Dentistry, University of Sheffield, Sheffield S10 2TA, UK; jwoodley1@sheffield.ac.uk (J.P.W.);

**Keywords:** fibroblast, 3D cell culture, biomaterials, electrospinning, scaffold

## Abstract

In vivo, quiescent fibroblasts reside in three-dimensional connective tissues and are activated in response to tissue injury before proliferating rapidly and becoming migratory and contractile myofibroblasts. When deregulated, chronic activation drives fibrotic disease. Fibroblasts cultured on stiff 2D surfaces display a partially activated phenotype, whilst many 3D environments limit fibroblast activation. Cell mechanotransduction, spreading, polarity, and integrin expression are controlled by material mechanical properties and micro-architecture. Between 3D culture systems, these features are highly variable, and the challenge of controlling individual properties without altering others has led to an inconsistent picture of fibroblast behaviour. Electrospinning offers greater control of mechanical properties and microarchitecture making it a valuable model to study fibroblast activation behaviour in vitro. Here, we present a comprehensive characterisation of the activation traits of human oral fibroblasts grown on a microfibrous scaffold composed of electrospun polycaprolactone. After over 7 days in the culture, we observed a reduction in proliferation rates compared to cells cultured in 2D, with low KI67 expression and no evidence of cellular senescence. A-SMA mRNA levels fell, and the expression of ECM protein-coding genes also decreased. Electrospun fibrous scaffolds, therefore, represent a tuneable platform to investigate the mechanisms of fibroblast activation and their roles in fibrotic disease.

## 1. Introduction

Fibroblasts are found in the stromal compartment of tissues throughout the body in a quiescent state and have roles in extracellular matrix (ECM) deposition and maintenance [1]. Fibroblasts ‘activate’ in response to tissue injury [2]; their activation is characterized by increased proliferation rates, ECM deposition, and differentiation to an alpha SMA-positive, contractile, myofibroblast phenotype [2]. Deregulated fibroblast activation has a pivotal role in fibrosis and hypertrophic scaring, as well as in the progression of certain cancers [3].

The ECM in which fibroblasts reside provides points of attachment for cells in three dimensions. For this reason, fibroblasts have been grown in 3D culture models for many years. We summarize the most relevant aspects of this research here, although our previous review provides a more complete evaluation of the studies which have shaped our current understanding of fibroblast behaviour in 3D cultures [4]. Since fibroblasts were first cultured in collagen hydrogels, it has been apparent that a traditional 2D culture can give rise to a fibroblast phenotype that is closer to an activated state, with rapid proliferation and the expression of detectable levels of the myofibroblast marker α-SMA. Quiescent fibroblasts are more accurately mimicked in collagen cultures [5,6].

Fibroblast behaviour was characterized in natural hydrogels composed of collagen and fibronectin, with work progressing to investigate how material mechanical properties and biological features such as integrin binding sites influence fibroblast behaviour. Collagen hydrogel contraction assays were a useful tool for the investigation of fibroblast mechanoregulation. When anchored, mechanical tension forms within gels as fibroblasts try to contact them. In free-floating gels, fibroblast motility causes contraction. Post-contraction gels are referred to as relaxed [7]. The phenotypic changes observed in relaxed gels include reduced proliferation and reduced collagen deposition [7]. Cellular proliferation in monolayer cultures is an anchorage that is dependent and acts via the MAPK/ERK pathway [8]. ERK phosphorylation is reduced in relaxed gels, suggesting that the same pathway may be involved in regulating proliferation in 3D gels [8]. The association of integrins with the Src homology 2 domain-containing adapter protein (SHC) was postulated as a possible point of ERK/MAPK pathway disruption [8] though no further investigation of the mechanism was completed.

Entering the 21st century synthetic polymer-based hydrogels overtook natural gels due to their greater tunability. Synthetic hydrogels were used to investigate the effects of substrate stiffness, cellular polarity, and morphology. Increasing gel stiffness was linked to fibroblast activation [9]. One hydrogel study took this observation further to isolate specific biomechanical properties and their interconnectivity [10]. In 2D cultures, attachments form in just one plane creating apico-basolateral polarity [10]. Smithmyer used layered hydrogels to create a sandwich ‘2.5D’ culture that allowed fibroblasts to spread, a feature lost during 3D gel encapsulation but which can still make attachments in 3D. By comparison, 2D gel culture myofibroblast differentiation was not significantly reduced in the 2.5D culture as it was in 3D. However, in 2.5D cultures, fibroblasts became less responsive to stiffness than in 2D or 3D cultures. This suggested that other features of the culture geometry, such as cell–cell interactions enabled by cell spreading, were of more significance in controlling activation than stiffness. The response to substrate stiffness was measured by quantifying YAP nuclear localization—a precursor to Alpha smooth muscle actin (α-SMA) expression [10]. This study indicated the scope and direction of research in the field with highly modifiable biomaterials enabling the breakdown of individual aspects in cell–environment interactions.

In recent years, a wide variety of modern biomaterials and fabrication techniques have been used to elucidate fibroblast behaviour. One of the most recent strategies for interrogating fibroblast behaviour was based on the combination of electrospun fibres with polymer hydrogels to mimic both the fibrous protein structures and the hydrated proteoglycans of the ECM [11]. The presence of electrospun fibres within the hydrogel matrix enhanced cell spreading and provided an alternative method to that of Smithmyer for reducing the cell encapsulation and cell rounding that is observed in traditional hydrogels [11]. Rac 1 and Rho A GTPases were shown to be crucial for fibre-mediated cell spreading and contact guidance. Cdc42 also contributed to myofibroblast differentiation in the model [11].

Studies into the mechanisms controlling fibroblast activation were most prevalent in hydrogel systems, but there has been limited progress in electrospun scaffolds. Studies tend to focus on quantifying specific aspects of behaviour in response to scaffold features. Examples include the relationship between the fibre diameter and cell viability and attachment [12] or the impact of aligned fibres on cell migration kinetics [13]. The inflammatory response in fibroblast cells has also been investigated [14,15], and one study was able to quantify changes to Rac GTPase activation [16]. To date, no study has reported a detailed analysis of fibroblast activation behaviour in an electrospun scaffold with the same focus on microarchitecture and mechanical properties as in the hydrogel system.

In this study, our goal was to fully characterise the features of fibroblast activation in a non-hydrogel system. Our scaffold facilitates cell spreading, whilst reducing the cellular apico-basolateral polarity seen in a 2D culture. In our electrospun models, we observed a reduction in fibroblast proliferation based on reduced Ki67 expression. Ki67 is a nuclear protein that is expressed by all actively proliferating cells but it is not expressed by cells in cell cycle phase G0 [17]. ECM deposition and α-SMA expression were also reduced as the cells entered a quiescent vivo-like state.

## 2. Materials and Methods

### 2.1. Scaffold Manufacture

Scaffolds were electrospun using a custom vertical set-up containing a PHD2000 syringe pump (Harvard Apparatus, Cambridge, UK) connected to an Alpha IV Brandenburg power source (Brandenburg UK Ltd., Dudley, UK). A medical grade polycaprolactone (C_6_H_10_O_2_)n (PCL) Purasorb PC 12 (Corbion, Amsterdam, NL, USA) viscosity 1.2 dL/g was dissolved in a solvent solution containing 92.66% dichloromethane (DCM) (Fisher Scientific, Loughborough, UK) and 7.34% dimethylformamide (DMF) (Fisher Scientific, Loughborough, UK) with a final PCL concentration of 15% (weight/volume) (*w/v*). A 1 mL syringe (Becton Dickinson, Reading, UK) was attached to a blunt 20 ga metal needle (Fisnar Europe, Glasgow, UK) positioned 17 cm away from the grounded metal collector. The metal collector was covered in wax-free baking parchment to facilitate scaffold storage and detachment. A total of 2 mL of the polymer solution was dispensed at a rate of 1.5 mL/h with a voltage of 21.08 kV. Scaffolds were manufactured at room temperature. Scaffolds were stored at room temperature in sealed bags with desiccant (Merck KGaA, Darmstadt, Germany). A cork borer with a diameter of 8 mm was used to cut scaffolds for SEM analysis, whilst a 12 mm borer was used to cut samples for culture with cells. Scaffolds were sterilized in 100% ethanol (EtOH) (Fisher Scientific, Loughborough, UK) for 15 min, then rinsed 3 times in PBS. The scaffolds were placed in 12 well plates (Greiner Bio-One, Gloucester, UK) for seeding and subsequent culture.

### 2.2. Scaffold Characterisation

Scaffolds were characterized by scanning electron microscopy (SEM), image analysis, mechanical testing, and mercury intrusion porosimetry (MIP). FOR MIP, an Autopore V 9600 (Micromeritics Instrument Corporation, Atlanta, USA) machine was used with Micro-Active software (Micromeritics Instrument Corporation, Atlanta, USA). Mercury temperature was 25.34 ℃, and the report range was from 0.1 to 61,000 psia. A sample mass of 0.0260 g was used. SEM was conducted at the Electron Microscope Facility at Sheffield University using a Vega3 SEM (Tescan-UK Ltd., Cambridge, UK). An Edwards S150B sputter-coater (Edwards Vacuum UK, Burgess Hill, UK) was used to gold coat samples (2 min total sputter time) prior to imaging. To characterise the fibre size, and surface porosity, scaffolds were fabricated on different days using the same electrospinning conditions. Samples were cut from separate locations within each scaffold, including the central and peripheral sites. To calculate the surface pore area all individual pores in the top layer of the scaffold were measured. The sum of all areas was calculated as a percentage of the total image area using the following equation: $\left(\frac{Surface\;pore\;area\;\left(\mu m\right)}{Image\;area\;\left(\mu m\right)}\right)\times 100$. A total of 628 pores were measured from 9 SEM images taken from 3 different scaffolds. The images were analysed using ImageJ v1.52p. Each sample was imaged in three areas with 270 fibres measured for the fibre diameter and angle. Fibre angles were normalized by subtracting the mean fibre angle for each scaffold from every individual fibre angle. This process rotated the mean fibre angle to zero across all samples and scaffolds. Fibre alignment was calculated as a percentage using the equation: $\left|1-\left(\frac{\left|mean-angle\right|}{90}\right)\right|$ × 100. To achieve a more accurate average percentage alignment value, angles that deviated by more than +/− 90° from the mean were corrected by adding 180°. This was necessary as, whilst fibres become less aligned in one axis (left to right) when +/− 90° from the mean, they became more aligned with the mean if measured from right to left.

For mechanical testing, 3 PCL scaffolds prepared on different days under the same conditions were tested using a Lloyd LRX universal testing machine (Lloyd Instruments Ltd., Bognor Regis, UK). A 50N load cell was fitted, and testing was conducted at room temperature with a crosshead speed of 10 mm/min. Each scaffold was used to produce 2 technical replicates of 70 mm × 10 mm × 0.25 mm, which were prepared to ASTM D882 standards where applicable. Thickness was measured using a Mitutoyo 293–816 digital micrometer (Mitutoyo UK Ltd., Halifax, UK) with a 0.001 mm resolution. Nexygen 4.1 software was used to obtain stress–strain curves from which the elastic modulus of each specimen was estimated. The sample stress (σ) was determined by the applied load (F) divided by the original cross-sectional area (A0); σ = A0/F. Change in gauge length (ΔL) divided by the initial gauge length (L0) gives unitary deformation or strain (ε = ΔL/L0). Consequently, the elastic modulus (E) can be calculated (E = σ/ε).

### 2.3. Cell Culture

Mouse embryonic NIH 3T3 fibroblasts (ATCC^®^ CRL-1658™) were acquired and used between passage numbers 10 and 40. Normal oral fibroblasts (NOF) were originally isolated and characterized as described in [18] and were kindly provided to us for this study by Dr. Helen Colley [19]. They were isolated from a buccal biopsy taken with written, informed consent (ethical approval number 09/H1308/66) and, in this study, were used between passage number 6 and 15. Tissue culture plastic (TCP) grown fibroblasts were used as a control for all experiments excluding fluorescent labelling and SEM imaging, where glass coverslips were used instead. 3T3 Fibroblasts were used in initial PrestoBlue and PicoGreen assays. NOF were used for all initial assays, Live/Dead imaging, Ki67 immunofluorescent staining, SEM imaging, and qPCR. Cells were cultured in high glucose Dulbecco’s modified Eagles medium (DMEM) (Sigma Aldrich, Gillingham, UK) supplemented with 10% fetal calf serum, 0.5% penicillin-streptomycin, and 0.5% L-glutamine (all Sigma Aldrich, Gillingham, UK). Media was stored at 4 °C and prewarmed before use. The cells were seeded onto scaffolds at a density of 10,000 cells in 10 μL of media. Scaffolds were pre-wetted with 800 μL of the during diffusion seeding. During bead seeding, the 10 μL of the cell-containing media was placed centrally onto the scaffold for 10 min prior to adding 800 μL of media. Senescence was induced in NOF and grown to 60% confluence using 500 μM H_2_O_2_ (Sigma Aldrich, Gillingham, UK) diluted in supplemented DMEM. 5 ng/mL TGF-β (R&D Systems, Abingdon, UK) in supplemented DMEM was used to stimulate fibroblasts 1 day after seeding on scaffolds or in 12 well plates (Greiner bio-one, Gloucester, UK). Fibroblasts were harvested for 3 or 6 days after stimulation with TGF-β (day 4 and day 7 post-seeding).

### 2.4. Fibroblast Viability

To quantify cell viability, a PrestoBlue™ (Invitrogen Thermofisher, UK) assay was used. Culture media was removed, and fibroblasts were incubated with 1 mL of 10% PrestoBlue reagent (Invitrogen, Thermo Fisher Scientific, Loughborough, UK), which was diluted in the culture media at 37 °C and 5% CO2 for 1 h. Plates were wrapped in foil during incubation, and to ensure minimal light exposure, all work was conducted in unlit hoods. After incubation, 200 μL of the PrestoBlue reagent from each sample was removed and placed in a 96-well plate in triplicate. Plates were read using an M200 plate reader (Tecan UK, Reading, UK) and Magellan V7.2 analysis software using an excitation wavelength of 550 nm and an emission wavelength of 590 nm.

A Live Dead™ viability/cytotoxicity kit (Thermo Fisher Scientific, Loughborough, UK) was also used to estimate cell viability. Samples were washed in PBS (Merck, Germany) two times before 500 mL of staining solution was added. The staining solution contained 0.05% calcein acetoxymethyl (AM) (Thermo Fisher Scientific, Loughborough, UK) and 0.2% ethidium homodimer-1 (Thermo Fisher Scientific, Loughborough, UK) in the culture media. Samples were incubated in the dark for 20 min at room temperature. An Axioplan 2 (Zeiss UK, Cambridge, UK) fluorescent microscope was used to image the samples.

### 2.5. Fibroblast DNA Quantification

A Quant-iT™ PicoGreen™ double-stranded DNA (dsDNA) Assay Kit (Fisher Scientific, Loughborough, UK) was used to estimate the cell number. After washing samples with PBS, scaffolds were placed in eppendorf tubes containing 1% Triton X-100 (Sigma Aldrich, Gillingham, UK) in distilled water. Control cells were adherent to the well plate and were removed by scraping before being added to Eppendorf tubes. Thereafter, the cells were rapidly freeze–thawed 3 times using −80 °C freezers. After the final thaw, 100 μL from each sample was added to a 96-well plate in triplicate. A standard curve was also generated using a lambda DNA standard (Thermo Fisher Scientific, Loughborough, UK). A total of 100 μL of PicoGreen solution (0.005% Picogreen concentrate (Thermo Fisher Scientific, Loughborough, UK) was diluted in a 5% tissue buffer (Fisher Scientific, Loughborough, UK) in dH_2_O) and added to each sample as well as the standard curve. Ninety-six well plates were kept in the dark and left to react for 5 min until they were read using an M200 plate reader (Tecan UK, Reading, UK), and V7.2 Magellan analysis software was used with an excitation wavelength of 480 nm and an emission wavelength of 520 nm.

### 2.6. Fibroblast Proliferation

The immunofluorescent detection of Ki67 was used to assess what proportion of fibroblasts were proliferative (Ki67+) or non-proliferative in cell cycle stage G0 (Ki67-). Fibroblasts were grown on glass coverslips or PCL scaffolds before being fixed with 4% paraformaldehyde (Alfa Aesar, Thermo Fisher, Heysham, UK) for 15 min. The samples were washed in PBS three times before staining commenced and were blocked in a blocking buffer (PBS, 5% donkey serum; Jackson ImmunoResearch, Cambridge, UK), 0.3% triton X-100 (Sigma Aldrich, Gillingham, UK)), for 60 min. The blocking buffer was removed, and the primary antibody (Cell Signaling Technology, Danvers, USA) was diluted 1:400 in a dilution buffer (PBS, 1% BSA (Sigma-Aldrich, Gillingham, UK), 0.3% triton X-100) was applied, and incubated overnight at 4 °C. The primary antibody was removed, and samples were washed three times in PBS for 5 min each. Samples were then incubated in a fluorochrome-conjugated secondary antibody (Cell Signaling Technology, Danvers, USA) diluted 1:1000 in a dilution buffer for 75 min. Samples were washed three times in PBS for 5 min before being applied to microscope slides with a drop of VECTASHIELD™ antifade mounting medium with DAPI (Vector Laboratories, Oxford, UK)). For F-actin staining, the cells were fixed and blocked, as described above. TRITC-conjugated Phalloidin (Merck Millipore, Burlington, USA) was used to visualize F-actin at a concentration of 1:1000 diluted in a blocking buffer alongside DAPI (Merck Millipore, Burlington, USA), which was diluted 1:2000. Samples were incubated for 45 min at room temperature before being washed 3 times in PBS. Samples were imaged using an Axioplan 2 (Zeiss UK, Cambridge, UK) fluorescent microscope and Image-Pro Plus software™. Cell counting was performed in ImageJ v1.52p.

### 2.7. Quantitative PCR

A Direct-zol RNA miniprep kit (Zymo Research Europe, Freiburg im Breisgau, DE, USA) was used for RNA extraction. RNA was isolated from the NOF grown on scaffolds and TCP for 1, 4, or 7 days with 3 repeats at each time point. Where no time dependence was observed, all values were used to calculate averages for graphs and statistics, giving an n value of 9. Induced senescent fibroblasts were grown for 7 days before RNA extraction. RNA was extracted from TGF-β stimulated fibroblasts after 4 or 7 days in culture with 3 repeats at each time point. Four scaffolds and 4 seeded wells were grouped for each sample to increase the RNA yield. Scaffolds were rinsed in PBS before TRI Reagent^®^ (Zymo Research Europe, Freiburg im Breisgau, DE, USA) and were used to dissolve scaffolds and lyse cells in an Eppendorf tube. TCP-grown cells were scraped with a pipette tip after the TRI Reagent was added to ensure cell detachment and lysis. Ethanol (100%) (Fisher Scientific, Loughborough, UK) was added to the Tri Reagent and mixed. Samples were transferred to a Zymo-Spin IICR Column (Zymo Research Europe, Freiburg im Breisgau, DE, USA) and centrifuged. Next, an RNA wash buffer was added to the tube and centrifuged before DNase 1 (6 U/μL), which was mixed with an added DNA digestion buffer. Samples were incubated at room temperature for 15 min and then washed twice with Direct-Zol™ RNA Prewash. One final wash using an RNA wash buffer was completed before RNA elution. RNA was eluted using DNase/RNase-Free water and centrifugation. RNA purity and concentration were estimated using the Nanodrop 1000 (Thermo Fisher Scientific, Loughborough, UK). A concentration of 250 ng/μL RNA in nuclease-free water (Invitrogen, Thermo Fisher Scientific, Loughborough, UK) was used for cDNA conversion. cDNA conversion was completed using a sample RNA mixed with an RT buffer, dNTPs, random primers, and a multiscribe^®^ enzyme (All reagents—Thermo Fisher Scientific, Loughborough, UK), plus nuclease-free water. Once mixed, the solution was added to a PCR Tube (VWR International, Radnor, USA) and heated in an AB 2720 thermal cycler (Applied Biosystems, Thermo Fisher Scientific, Loughborough, UK) for 10:00 at 25 °C, 120:00 at 37 °C, and 5:00 at 85 °C. cDNA was then stored at −80 °C before being used in qPCR. TaqMan^®^ gene expression assays (Thermo Fisher Scientific, Loughborough, UK) were used to quantify the mRNA expression of senescence-associated genes CDKN2A (P16) (Hs00923894_m1), CDKN1A (P21) (Hs00355782_m1) and IL6 (Hs00985639_m1), cytoskeletal genes VIM (Hs00958111_m1) and ACTA2 (α-SMA) (Hs00426835_g1) and ECM coding genes FN1 (Hs01549976_m1), COL1A1 (Hs00164004_m1), COL3A1 (Hs00943809_m1), and VCAN-V1 (Hs01007937_m1). For qPCR, each strip tube (Qiagen, Venlo, NL, USA) contained 5 μL of qPCRBIO probe blue mix Lo-ROX (PCR Biosystems Ltd. London, UK) with 0.5 μL B2M control mix, 0.5 μL TaqMan^®^ target gene probe (Applied Biosystems, Thermo Fisher Scientific, Loughborough, UK) and 3 μL nuclease-free water (Invitrogen, Thermo Fisher Scientific, Loughborough, UK). A Rotor-Gene Q 2plex (Qiagen, Venlo, NL, USA) was used for real-time PCR (qPCR). A two-step cycling profile was used with a 10 min hold at 95 °C followed by 40 Cycles consisting of 45 s at 60 °C followed by 10 s at 95 °C. Green (excitation 470 nm ± 10, detection 510 nm ± 5) and yellow (excitation 530 nm ± 5, detection 557 nm ± 5) fluorescence were detected with a gain of 5. The software version used was Rotor-Gene Q Software 2.3.1.49.

### 2.8. Fibroblast Morphology

To study cell morphology on glass coverslips and electro-spun scaffolds, the samples were fixed and then dehydrated. First, culture media was removed, and samples were washed with PBS twice for two minutes. Then, cells were fixed using 2.5% glutaraldehyde (Merck KGaA, Darmstadt, Germany) in PBS for 1 h. The glutaraldehyde solution was removed, and samples were washed 3 times for 15 min with PBS. Next, the samples were washed in distilled water for 5 min before sequential dehydration using ethanol (Sigma Aldrich, Gillingham, UK). Samples were placed in increasing concentrations of ethanol in distilled water for 15 min each from 35% (*v/v*) to 60%, 80%, 90%, and 100%. Next, the samples were treated with ethanol: hexamethyldisilazane (HMDS) (Sigma Aldrich, Gillingham, UK) 1:1 (*v/v*) solution for 1 h. Finally, samples were treated twice with 100% HMDS for 5 min before they were left to air-dry for 1 h prior to gold-coating (Edwards S150B sputter-coater) and SEM imaging (Tescan, Vega3).

### 2.9. Statistics

All statistics were conducted using GraphPad Prism version 9.1.1 unless otherwise stated. Where relevant, the *p*-value ranges and biological and technical replicate values are included in figure legends. A Gaussian non-linear regression was used alongside the D’Agostino and Pearson normality test to visualize fibre diameter distribution. Unpaired t-tests with Welch’s correction were used to compare the results between different fibroblast culture environments unless otherwise stated. Pie charts were used to visualize the average proportion of cells that were proliferative or quiescent as a percentage of the total population, and actual values are listed in the text. QPCR values were converted to fold changes, with the control given in each qPCR figure, using the 2^−(ΔΔCT) method [20]. Graphs display the converted mean ΔΔCT values and standard deviation error bars calculated from each individually converted repeat. For statistical analysis, Dunnett’s T3 multiple comparisons test was conducted on unconverted ΔΔCT values with *p*-values displayed on the graphs.

## 3. Results

### 3.1. Scaffold Characterisation

**Figure 1:**
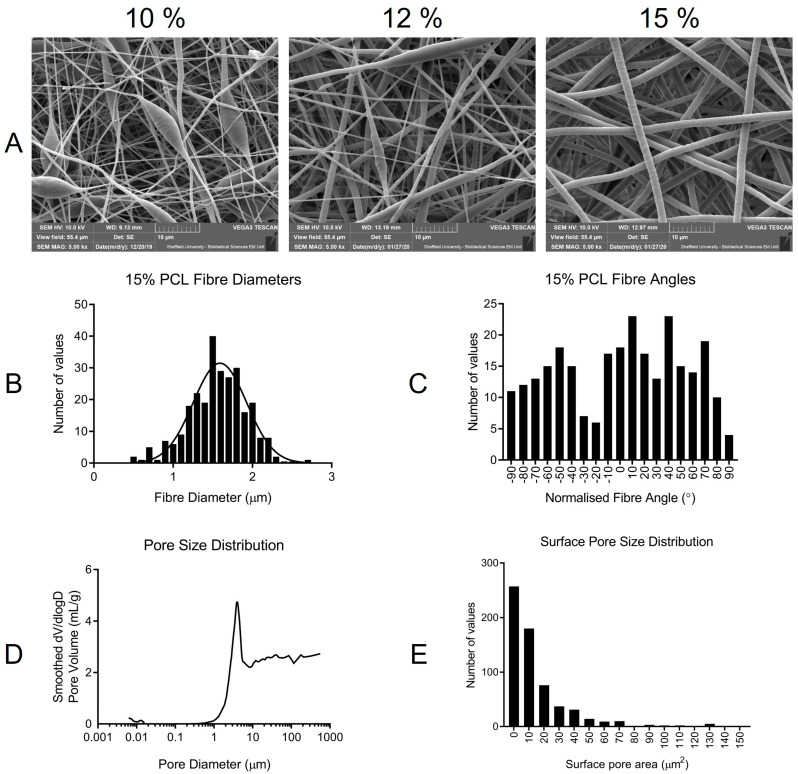
(**A**) Representative SEM images of 10, 12, and 15% (*w/v*) PCL electrospun scaffolds. Beading is evident at 10% and fibres vary in diameter at 12% before homogenizing at 15%. Further characterisation was only completed with 15% (*w/v*) scaffolds. (**B**) Fibre diameters are normally distributed according to the D’Agostino and Pearson normality test, and a Gaussian non-linear regression was plotted. (**C**) Fibre angle distribution. For angle and diameter, 10 fibres were measured in each of 27 images taken from 9 samples and isolated from 3 scaffolds (biological replicates = 3, technical replicates = 3). (**D**) Mercury Intrusion Porosimetry. Pore diameter distribution was plotted from intrusion data using GraphPad Prism. Only one sample was used for intrusion data (**E**) Surface pore area distribution (biological replicates = 3, technical replicates = 3).

The PCL concentration was determined to be optimal at 15% (*w/v*) PCL due to the ease of manipulation and homogeneity of the fibre diameter (Figure 1A). Once the ideal polymer concentration was identified, three scaffolds were fabricated and characterized to ensure inter-scaffold consistency before proceeding to cell culture experiments.

To determine the fibre diameter and angle, SEM images were analysed using ImageJ version v1.52p. The average fibre diameter was 1.56 μm (Figure 1B). Fibre diameters were normally distributed, according to the D’Agostino and Pearson test (*p* = 0.178). Fibre angles were spread evenly across the full range of possible angles (Figure 1C). The angles were not normally distributed according to the D’Agostino and Pearson normality test (*p* < 0.0001), indicating that the fibres were randomly orientated within the scaffold.

Scaffold porosity was calculated using two methods mercury intrusion porosimetry (MIC) and SEM image analysis using ImageJ software. The smallest pores began in the region of 0.8 μm in diameter (Figure 1D), and the total porosity was 74.5%. Bulk density was measured at 0.113 g/mL, with the apparent (skeletal) density at 0.442 g/mL. The technique has previously been used in electrospun scaffolds, although it has been suggested that flexible scaffolds may distend, causing fibres to bend and altering porosity readings [21]. To complement mercury intrusion data, surface porosity was calculated from SEM images. Surface porosity characterizes the surface topography with which fibroblasts interact upon seeding. The average surface porosity was 46%: a lower value than the mercury intrusion estimate. This variance occurs as SEM-based calculations only account for the pore and total image areas (μm^2^) without considering the volume (μm^3^) or depth of the pores. The distribution of these pore sizes is displayed in (Figure 1E). Most pores were small, ranging from 1 to 60 μm^2^. A small number of large pores, up to 150 μm^2^, were also observed. Mechanical testing of the 15% PCL electrospun scaffold revealed an average elastic modulus of 4.07 ± 0.71 MPa, an ultimate tensile strength of 0.82 ± 0.17 MPa, and elongation at a break of 190.9 ± 77.1%.

### 3.2. Assessment of Fibroblast Viability on Electrospun Scaffolds

Once scaffolds had been characterized, several assays were used to assess the viability and proliferation of two fibroblast types, 3T3 and NOF, on the scaffold. These included metabolic assays PrestoBlue and Live Dead as well as a double-stranded DNA (dsDNA) quantification method—PicoGreen.

**Figure 2:**
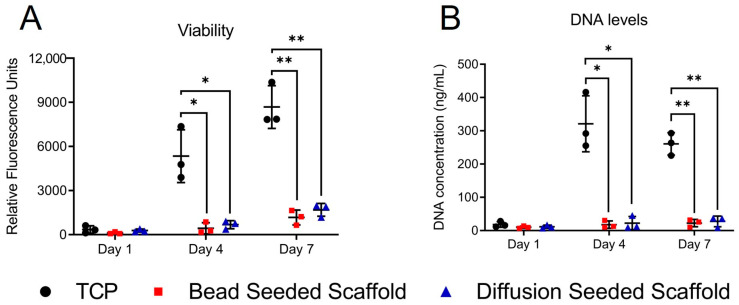
PrestoBlue™ and PicoGreen™ assay data using 3T3 fibroblasts. (**A**) PrestoBlue Viability data (**B**) PicoGreen data. Fluorescence and absorbance were read using a Tecan M200 plate reader and Magellan analysis software. GraphPad Prism software was used to generate graphs. ** *p* < 0.01, * *p* < 0.05. Error bars represent the standard error, (biological replicates = 3, technical replicates = 3).

Significantly, more metabolically active cells were present on the TCP than on the scaffold at day 4 and 7 post-seeding, regardless of the scaffold seeding technique, as assessed by PrestoBlue (Figure 2A) and PicoGreen data (Figure 2B). From day 1 to 7, there was a significant increase in 3T3 TCP viability (*p* = 0.009) and dsDNA levels (*p* = 0.004). The modest increase in viability and dsDNA levels from day 1 to 7 on the bead-seeded scaffold was not statistically significant. However, the increase in viability for diffusion-seeded scaffolds was significant (*p* = 0.025), with some proliferation taking place. The cells reached confluence between days 4 and 7 on the TCP (microscope observation not shown), explaining the reduced growth rate observed from day 4 to day 7 in Figure 2B.

**Figure 3:**
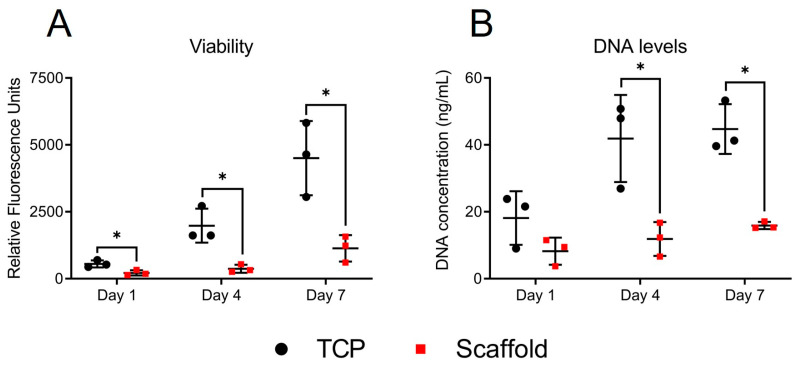
PrestoBlue™ and PicoGreen™ assay data using NOF. (**A**) PrestoBlue viability data (**B**) PicoGreen data. Fluorescence and absorbance were read using a Tecan M200 plate reader and Magellan analysis software. GraphPad Prism software was used to generate graphs. * *p* < 0.05. Error bars represent standard deviation, (biological replicates = 3, technical replicates = 3).

NOF represented a more relevant model than the immortalized mouse embryonic cell line 3T3 and was thus used to validate the observations using 3T3 cells. Only the diffusion seeding technique was used in NOF experiments. Once again, PrestoBlue viability and PicoGreen dsDNA quantification assays showed a reduction in the number of cells on the scaffold (Figure 3). There were a significant increase in both NOF TCP viability (*p* = 0.038) and dsDNA (*p* = 0.014) levels from days 1 to 7. The increase in NOF viability and dsDNA levels, when grown on the scaffold, was not significant.

**Figure 4:**
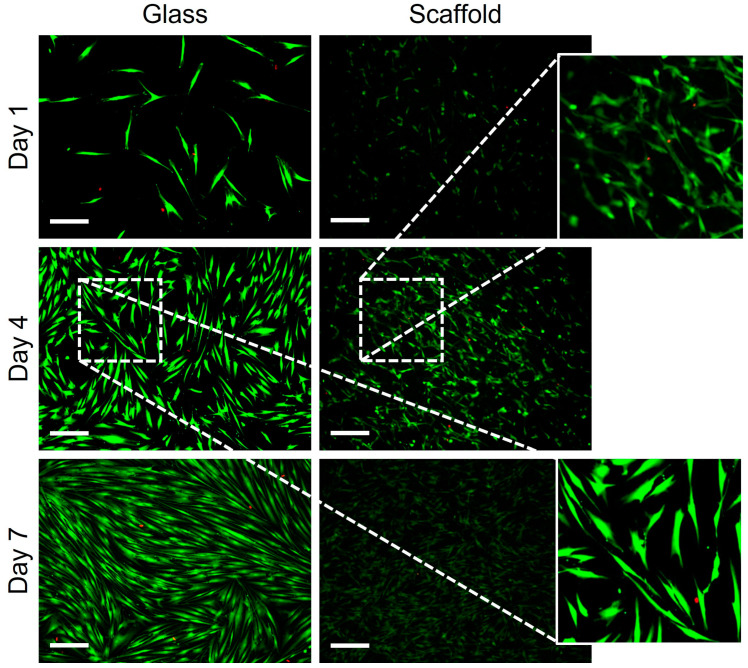
Representative live dead fluorescent images taken at day 1, 4, and 7 during the post seeding of normal oral fibroblasts onto PCL scaffolds and glass cover slips. The same image enhancement using ImageJ software to subtract background fluorescence and increase brightness was used for all images. Live cells are stained green and dead cells are stained red. Scale bar (white) is equal to 200 μm.

To assess whether the reduction in growth rate was due to cell death on the scaffolds, a Live Dead™ assay was used to stain the live fibroblasts green using Calcein AM and dead cells red using ethidium homodimer 1. No change in cell death on the scaffold was observed compared to fibroblasts grown on a 2D glass coverslip control (Figure 4). This suggests that rather than excessive cell death, reduced proliferation rates in the NOF grown on our PCL scaffolds were responsible for the reduced growth rate observed (Figure 2 and Figure 3).

**Figure 5:**
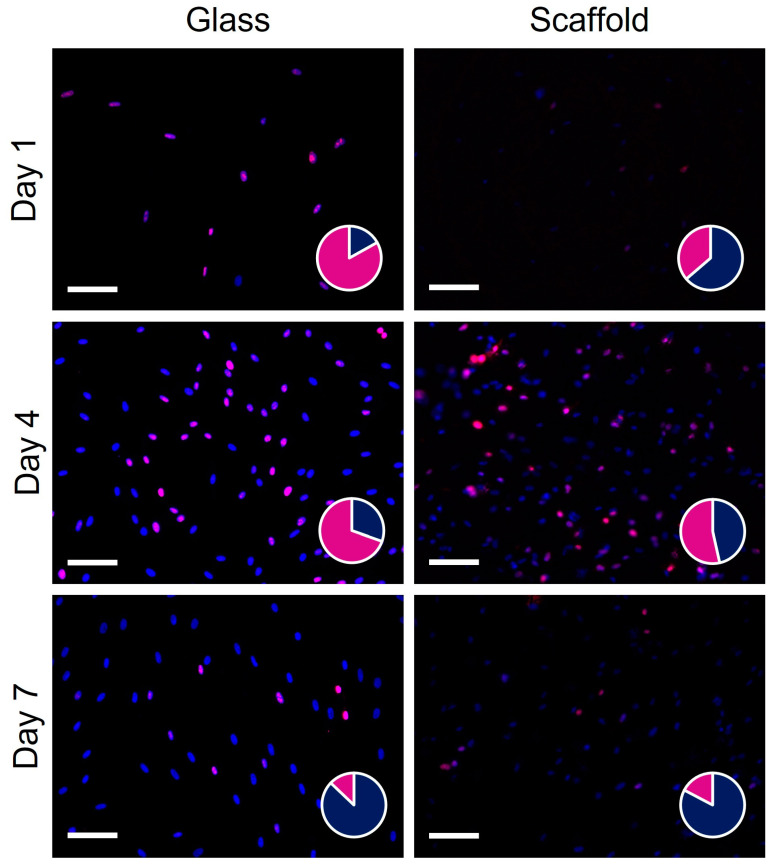
Representative Ki67 immunofluorescent images. The same image enhancement using ImageJ software to subtract background fluorescence and increase brightness was used for all images. Ki67 expressing NOFs were visualized with a CY3 conjugated secondary antibody and appeared purple in the merged images. All cell nuclei were counter stained with DAPI. Superimposed pie-charts show the proportion of cells which were proliferative (Ki67+/DAPI+) in purple and the proportion that were quiescent (Ki67-/DAPI+) in blue. Pie charts were generated after counting all the cell nuclei from 3 randomly positioned images taken from 3 technical replicates from each of the 3 biological replicates for a total of 27 images per time point. Scale bar (white) is equal to 100μm.

Live dead microscopy also revealed a difference in fibroblast morphology on the scaffolds. The fibroblasts appeared smaller and were either rounded in morphology or demonstrated long slender projections when grown on electrospun scaffolds (cutout). The proportion of rounded cells appeared to fall, and the proportion of elongated spindle-like fibroblasts increased from day 1 to 4 and day 4 to 7, although this has not been quantified. Fibroblasts grown on glass coverslips have a classic morphology with a broader and more spread cytoplasm (cutout). As cell death was not responsible for the reduced fibroblast growth on the PCL scaffold, we proceeded with a cell cycle assay to test for changes in proliferation rates (Figure 5).

### 3.3. Evaluation of NOF Proliferation on Electrospun Scaffolds

Live dead imaging suggested that cell death was not responsible for the fall in growth rates observed on electrospun scaffolds. For this reason, Ki67 immunofluorescence was selected as a method with which to visualize and quantify proliferating cells. Ki67 is expressed in all cells that are active in the cell cycle apart from those in phase G0: the quiescent cell population [17]. At day 1, there were significantly less (*p* < 0.0001) proliferative cells on PCL scaffolds (36%) compared to the glass (83%). By day 4, some areas of the glass were beginning to approach confluence, and there was a small reduction in the number of proliferative cells (70%). On the scaffold, fibroblasts became more proliferative (54%), perhaps as cells became more accustomed to the 3D environment. There were still significantly less proliferating cells on the scaffold than on the glass (*p* = 0.0002). By day 7, the proportion of proliferating cells had decreased on both the glass (13%) and scaffolds (17%): this was likely the result of cells reaching confluence.

The number of cells per image reflected the changes in the proportion of proliferating cells. On the glass, there was a rapid rise in the cell number from an average of 16 cells from day 1 to 99 on day 4, but there was no clear change between days 4 and 7, by which time the average number of cells per image was 94. Cells may continue to grow or spread between days 4 and 7, causing a slight reduction in cells per image on day 7. On scaffolds on day 1, there was a greater number of NOF, with 45 cells per image. There was modest growth between days 1 and 4 (45 to 87) and again from days 4 to 7 (87 to 123).

### 3.4. Assessing the Cause of Low Proliferation Rates in Scaffold Grown NOF

**Figure 6:**
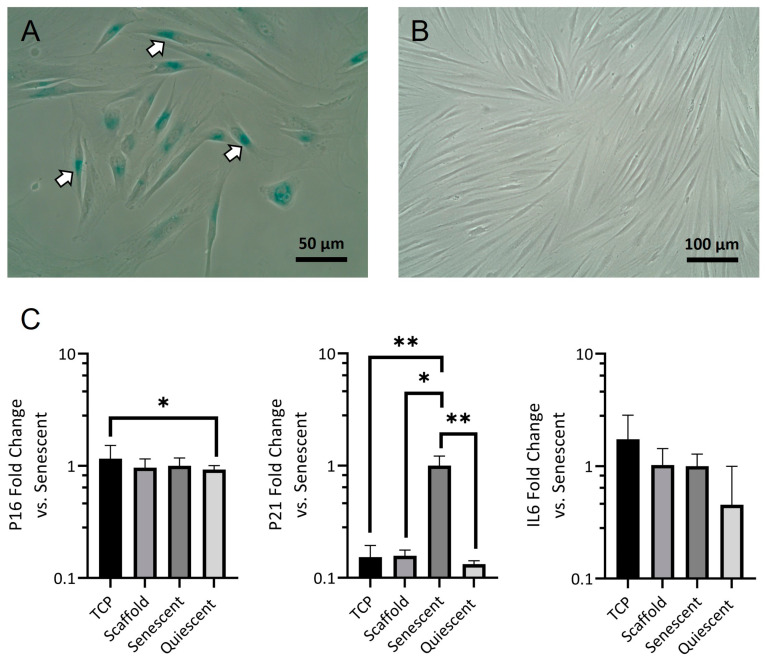
Further analysis of the low proliferation state observed in scaffold grown NOF. (**A**) Shows representative β-gal staining in H_2_O_2-_treated NOF with arrows indicating some of the β-gal-stained cells. (**B**) NOF grown to confluence as a model of quiescence. (**C**) Graphs displaying qPCR results for senescence associated genes P16, P21, and IL6. Fold changes in gene expression are ‘vs’ senescent NOF and are presented on a logarithmic axis. ** *p* < 0.01, * *p* < 0.05. Error bars represent standard deviation. TCP and scaffold (biological replicates = 9, technical replicates = 3), Senescent and Quiescent (biological replicates = 3, technical replicates = 3).

To determine whether Ki67 downregulation was the result of cellular quiescence or senescence, we screened several senescence-associated genes using qPCR analysis. To begin, we induced senescence and quiescence in NOF populations to function as the controls. Figure 6A shows representative β-gal staining in induced senescent NOF, and Figure 6B shows confluent NOF, which was used as a model of quiescence. QPCR gene expression analysis revealed no significant difference in the levels of P21, P16, or IL6 mRNA between the scaffold and TCP-grown NOF (Figure 6C). In H_2_O_2_ induced senescent NOF, we saw a statistically significant rise in P21 expression compared to the scaffold and TCP-grown NOF, as well as in NOF-grown to confluence in a T75 flask. As such, the fall in Ki67 levels (Figure 5) and in scaffold-grown NOF was likely to be indicative of a quiescent phenotype rather than a senescent one.

### 3.5. Fibroblast Morphology

**Figure 7:**
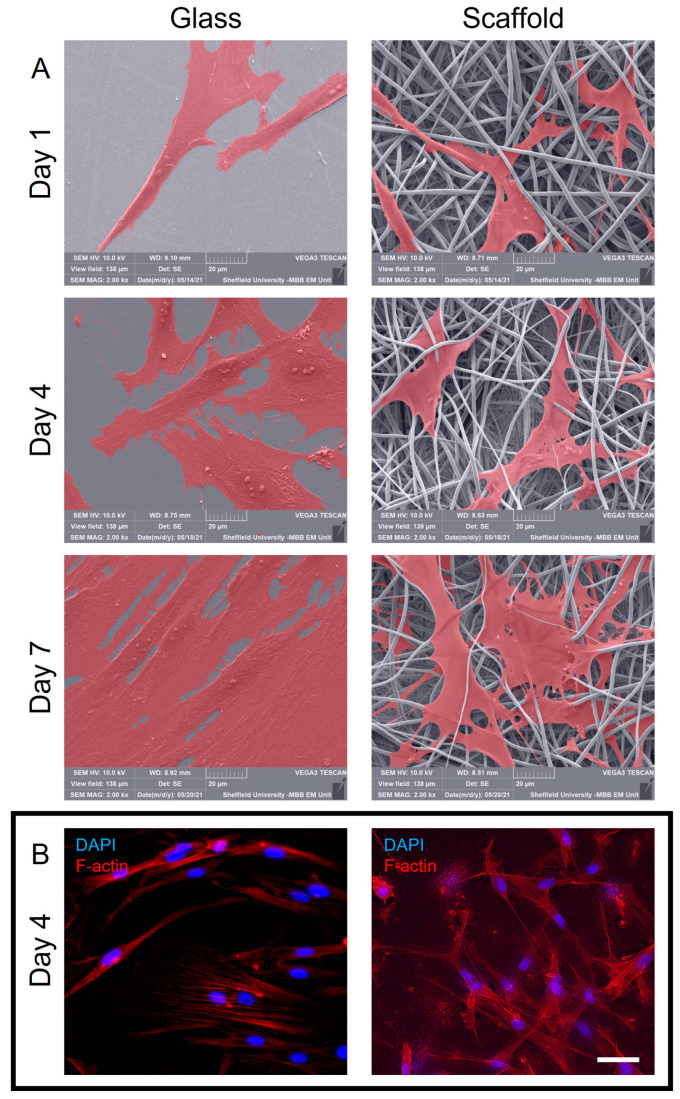
(**A**): Representative SEM images showing NOF morphology on glass cover slips and electrospun scaffolds. Images are magnified 2000× and scale bars are included with each image. Fibroblasts have been artificially coloured red using Adobe Photoshop 2021. (**B**): F-actin (red) immunofluorescent staining and DAPI (blue) staining of NOF grown on TCP and scaffolds. The scaffold grown fibroblasts were imaged using a z-stack; TCP is a single focus image. Scale bar = 50 um.

To gain a better understanding of fibroblast morphology and attachment to the fibrous scaffold, high-magnification images were captured using SEM (Figure 7A). Fibroblasts are well spread across the surface of the scaffold, with some cells penetrating just below the first few fibres. Cells make attachments to multiple fibres with morphology dictated by the local fibre alignment and distribution. The multi-level nature of the scaffold fibres means that fibroblasts often make attachments to fibres both above and below them, particularly when the cell has penetrated deeper into the scaffold. There is no obvious change in morphology from day 1 to day 7, although cell density visibly increases. On glass coverslips, fibroblasts appear to coat the entire surface, whilst some areas of the scaffold were still visible between cells on day 7. Figure 7B shows immunofluorescent stained NOF grew on TCP and electrospun scaffolds. F-actin stress fibres appeared in both culture conditions. Fibroblasts exhibit more spreading with larger cell bodies when grown on TCP.

### 3.6. Fibroblast and Myofibroblast Associated Gene Expression

**Figure 8:**
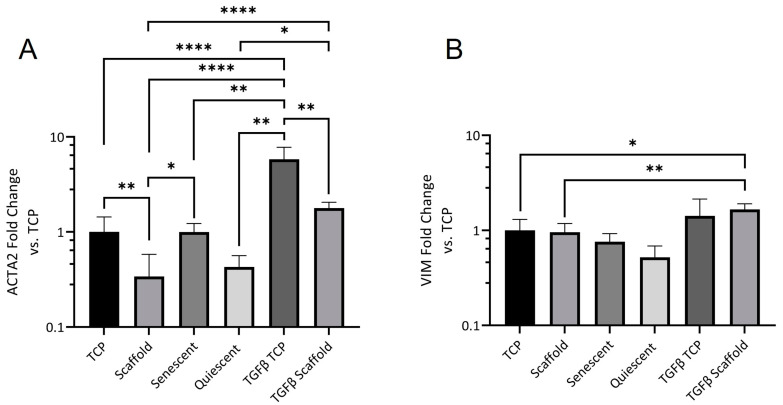
Graphs displaying qPCR results for cytoskeletal genes (**A**) ACTA2 and (**B**) VIM. Fold changes in gene expression are ‘vs’ TCP NOF and presented on a logarithmic axis. Comparison bars indicate significant differences: * *p* < 0.05, ** *p* < 0.01, **** *p* < 0.0001. Error bars represent standard deviation. TCP and scaffold (biological replicates = 9, technical replicates = 3), Senescent and Quiescent (biological replicates = 3, technical replicates = 3). TGFβ TCP and Scaffold (biological replicates = 6, technical replicates = 3).

VIM and ACTA2 are genes that encode the cytoskeletal proteins vimentin and α-SMA, respectively. Vimentin is expressed in both fibroblasts and myofibroblasts, whilst α-SMA is only expressed in myofibroblasts. ACTA2 expression was significantly reduced (*p* = 0.0046) in scaffolds cultured with fibroblasts, indicating a reduction in the number of activated cells (Figure 8A). TGFβ stimulation yielded a significant increase in ACTA2 expression for both TCP and scaffold-grown fibroblasts. Despite the increase in expression, TGFβ stimulated fibroblasts that were cultured on scaffolds still displayed significantly less ACTA2 expression than TGFβ stimulated fibroblasts grown on TCP. VIM expression was not significantly different between TCP or scaffold-grown fibroblasts, although expression showed a modest increase after TGFβ stimulation (Figure 8B).

### 3.7. ECM Gene Expression

**Figure 9:**
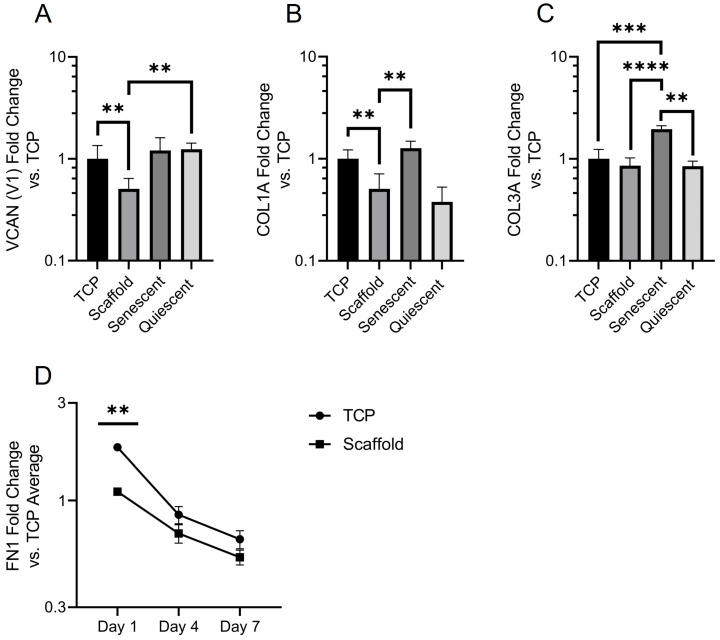
Graphs displaying qPCR results for Extracellular matrix genes. (**A**) Relative VCAN (V1), (**B**) COL1A, and (**C**) COL3A expression. (**D**) FN1 expression at day 1, 4, and 7. Fold changes in gene expression are ‘vs’ TCP NOF and are presented on a logarithmic axis. Comparison bars indicate significant differences: ** *p* < 0.01, *** *p* < 0.001, **** *p* < 0.0001. Error bars represent standard deviation. A-C: TCP and scaffold (biological replicates = 9, technical replicates = 3), Senescent and Quiescent (biological replicates = 3, technical replicates = 3). (**D**) Per day (biological repeats = 3, technical repeats = 3).

To further investigate fibroblast activation traits, qPCR was used to quantify the expression of four ECM constituents that were known to be expressed by activated fibroblasts [22]. Versican expression was significantly lower in scaffold-grown NOF than TCP-grown NOF and confluence-induced quiescent NOF (Figure 9A). Collagen type 1 expression was significantly reduced in scaffold-cultured fibroblasts when compared to both TC- cultured and senescent fibroblasts. There was no difference in collagen type 1 expression between quiescent fibroblasts and those grown on the scaffolds (Figure 9B). Collagen type 3 expression was equal in all samples apart from senescent fibroblasts, in which its expression was elevated (Figure 9C). Finally, unlike the other ECM constituents tested, fibronectin expression was shown to be time-dependent, with expressions in both conditions decreasing over time (Figure 9D). There was significantly more fibronectin expression at day 1 in TCP-grown fibroblasts than those grown on scaffolds.

## 4. Discussion

Fibroblasts exhibit a distinct behaviour depending on the physical and chemical properties of the medium on which they are cultured. Our goal was to produce polycaprolactone scaffolds to characterise fibroblast activation behaviour in a biomimetic environment.

Beading, coupled with poor physical properties during handling and wetting, led to our choice to halt the production of 10 and 12% PCL scaffolds. Enhanced handling alongside homogenous fibre diameters throughout the scaffold made 15% PCL the ideal concentration. Interestingly using another PCL brand (Sigma Aldrich) (average MW—80,000) at 10% concentration produced scaffolds that resembled (data not included) the 15% PCL (Purasorb—PC12) scaffolds, highlighting the sensitivity of the electrospinning process. Purasorb PCL was used as it is a medical-grade polymer. With future clinical applications in mind, optimizing the model from the outset of the project was logical. The presence of beading has been attributed to instability in the electrospinning process [23] with solution viscosity, surface tension, and electrical charge contributing to the appearance of beading [12]. Chen et al. observed that the electrospinning of fibres at large diameters could become bimodal. The normal distribution of fibre diameters was important when characterizing fibroblast behaviour, as cellular interactions with fibres of different diameters have been shown to vary [12]. The effect of the fibre size is one feature that has been previously investigated using electrospun scaffolds [12]. Chen et al. observed that fibroblasts attached and proliferated best on fibres, with an average diameter of 428 nm. As fibre diameter increased up to 1051 nm, both proliferation and attachment profiles were reduced. Interestingly, at 117 nm, fibres formed with beads, cell proliferation, and attachment were greatly reduced. At the opposite end of the spectrum, in scaffolds with an average fibre diameter of 1.647 μm, adhesion and proliferation increased, though not to the level observed on 428 nm fibres. With an average fibre diameter of 1.56 μm, it is worth noting that we did not observe a bimodal fibre distribution as seen by Chen et al. at a very similar average fibre diameter of 1.647 μm [12].

Fibre alignment in electrospun scaffolds has been shown to influence cellular alignment and gene expression [24]. Aligned fibres may lead to the deposition of aligned ECM proteins and could have clinical applications in wound healing and tissue regeneration [13]. We chose to work with randomly aligned fibres to mimic the random orientation of the buccal tissue from, which NOF were derived [25]. Porosity is another important feature of 3D culture platforms. Porosity in polymer hydrogels has been shown to influence cell behaviour, particularly fibroblast motility [26,27]. Several techniques have been used to assess electrospun scaffold pore sizes, including capillary flow porometry, liquid extrusion, and mercury intrusion porosimetry [21]. Capillary flow porometry can only provide values for the mean flow and pore diameter, not the pore size distribution or porosity. Of liquid and mercury porosimetry, mercury is the favoured technique as it has the potential to provide a more accurate estimation of pore sizes [21]. However, the high pressure required to force mercury through the pores of electrospun scaffolds can lead to scaffold deformation. Rutledge et al. proposed a correction technique. Corrected porosity values for PCL scaffolds of varied weight percentages and solvent compositions fell between 76 and 87% [28]. Our uncorrected porosity value of 75% was close to this range. Without using identical solvent and spinning systems or attempting to correct our porosity value, it is difficult to assess how accurate our porosity value is or how effective the Rutledge correction technique may be. Pore sizes between 0 and 130 μm^2^ enable fibroblasts to attach to multiple fibres and spread across multiple pores. A scaffold with pores significantly larger than a cell body results in NOF, which elongates along single fibrous struts. Finally, we have reported whole-scaffold mechanical properties. With an average elastic modulus of 4.07 MPa, the 15% PCL electrospun scaffold was stiffer than native soft tissues of the body which present elastic moduli in a range from 1 to 50 KPa [29]. Our PCL membrane was less stiff than TCP, which has an elastic modulus of approximately 1 GPa. Whilst these values are useful to compare inter-scaffold mechanical and handling properties, evaluating the impact of scaffold stiffness on fibroblast behaviour through the use of macro-mechanical properties would be inappropriate. Fibroblasts receive their cues from a small area of the scaffold with the stiffness of individual fibres and a more appropriate measure of material mechanical properties; for example, atomic force microscopy (AFM) is a technique that has the ability to measure the properties of fibres approaching the nanometer scale [30]. The studies highlighted to indicate the importance of thorough scaffold characterization, particularly in terms of fibre size, porosity, and alignment, as well as showing the versatility of the electrospun scaffold.

### Activation Behaviour

Having characterized our scaffold, we began to evaluate aspects of fibroblast activation behaviour, beginning with fibroblast proliferation. The reduction in viability observed in the PrestoBlue assay was due to reduced cell numbers, as shown by a reduction in dsDNA levels. Both 3T3 fibroblasts and NOF behaved in a comparable manner in viability studies; however, we chose to proceed with only NOF for further studies. As primary cells, NOF represents a more relevant cell when considering activation behaviour and its role in human disease than immortalized non-human cell lines such as 3T3 or L929 mouse fibroblasts [31]. Despite working with primary fibroblasts, we must acknowledge the impact of fibroblast heterogeneity on cellular behaviour. Fibroblasts derive from unique embryonic tissues and serve a variety of tissue-specific functions [32,33]. Even within different regions of the same tissue, dermal fibroblasts have displayed different morphology, physiology, and function [34].

Live dead staining was used to evaluate whether the fall in the number of viable cells on the electrospun scaffold was due to an increase in cell death. There was no evidence of significant cell death on the electrospun scaffold. This suggested that a reduction in the proliferative capacity of the scaffold-grown fibroblasts was responsible for the fall in cell number. To evaluate the proportion of proliferative cells, we employed KI67 immunofluorescent staining. Despite a slower rate of growth, NOF appeared to be able to grow to a higher confluence on scaffolds than on glass coverslips. This is likely the result of differences in cell morphology. On the glass, fibroblasts display broad, well-spread cytoplasm, whilst on scaffolds, fibroblasts are smaller and have long slender projections or appear rounded. Despite the reduced proliferation rates and a higher proportion of quiescent cells, NOF reached confluence by day 7. As Ki67 downregulation has been observed in non-cycling cells of both a quiescent and senescent nature [35], we performed a qPCR screen for senescence-associated genes p21 and p16 [36]. We also quantified IL6 expression, an inflammatory cytokine associated with the senescence-associated secretory phenotype (SASP) [37]. We used H_2_O_2_ to induce oxidative DNA damage in fibroblasts. When used at sub-lethal concentrations, H_2_O_2_ can induce cellular senescence [38]. These cells served as our senescent control. We also used NOF grown to confluence as a model of quiescence [39]. Our results suggest that senescent control cells were at an early stage of senescence, where cells were β-Gal positive, and p21 was highly expressed, and before p16 expression became dominant and a stable senescent phenotype could be established. This phased expression of p21, followed by p16, has been observed in cells approaching and reaching replicative senescence [40]. Critically we saw no difference in the levels of any of the senescent markers between TCP and scaffold-grown fibroblasts or between scaffold-grown fibroblasts and the confluent quiescent cells.

After confirming that the reduced fibroblast numbers on electrospun scaffolds were the result of a higher proportion of quiescent non-proliferating cells, we investigated fibroblast morphology. SEM images proved difficult to interpret, with no obvious similarity to the fibroblast morphologies observed during live-dead imaging. In live dead images, NOF grown on the scaffolds appeared to be either rounded or elongated with thin bipolar projections, whilst those grown on TCP exhibited larger, though still mostly bipolar, cell bodies. Calcein AM was converted to the highly fluorescent calcein by intracellular esterase activity [41] and could, therefore, provide fluorescence across the entire cell. Conversely, in SEM images, scaffold-grown fibroblasts appeared spread with stellate projections where attachments were formed with multiple fibres. These projections may be primarily composed of ECM proteins, probably in the form of focal adhesions attached to deposited fibronectin. Such an extracellular protein would not be detected by intracellular calcein staining. Distinguishing fibroblasts from what may have been deposits of ECM was not easy in grayscale SEM images. We, therefore, reverted to immunofluorescent staining to gain a better appreciation of cell morphology. F-actin staining revealed a morphology that more closely mimicked that of NOF when observed on days 4 and 7 in live dead images.

Alpha-SMA is part of the contractile apparatus in myofibroblasts and its expression is widely regarded as a key indicator of fibroblast activation and differentiation [42,43]. The reduction in scaffold ACTA2 expression was rescued by TGFβ stimulation; however, the level of expression was still significantly lower than in stimulated NOF grown on TCP indicating that other aspects of the scaffold culture environment contribute to fibroblast activation control.

Finally, we characterized the expression of ECM genes. Versican is a large proteoglycan, and the V1 isoform has been shown to modulate cell cycle progression and enhance proliferation [44]. It is unsurprising then that versican expression was reduced in electrospun scaffold-cultured NOF. Versican binds to several cell surface receptors, including integrins and an epidermal growth factor receptor, which are both known to regulate proliferation [45]. Scaffold NOF also expressed significantly less versican than quiescent cells, showing that there is a difference in the mechanism of quiescence between contact-inhibited and scaffold-grown NOF. Collagen deposition is another hallmark of fibroblast activation [2]. Collagen 1A expression was significantly lower in scaffold-grown fibroblasts, although it was also reduced in contact-inhibited NOF. Collagen 3A expression was equal across the culture conditions except for in senescent fibroblasts, where it was significantly elevated. Whilst the involvement of collagens in senescence has been established there is little substantiated evidence of a role for collagen 3 in cellular senescence [46]. The time dependence of fibronectin deposition may provide an explanation for the higher proportion of rounded cells that were observed on scaffolds on day 1 in live dead images. Rapid fibronectin deposition provides a more amenable surface for fibroblast adhesion, and cell spreading occurs. This dependence of shape on fibronectin deposition is not seen in TCP-grown NOF, possibly due to the optimized cell adhesion surface of TCP. The fall in FN1 expression is likely due to cells becoming more confluent towards day 7, as the confluent cells used in Figure 6B showed a ΔΔCT value of 0.43 (data not shown): less than half of the average ΔΔCT value for TCP-grown scaffolds and much closer to the ΔΔCT values of TCP and Scaffold NOF at day 7—0.65 and 0.53, respectively.

It is important to acknowledge the differences between the TCP control and our scaffolds. Notably, the surface properties of TCP, including charge, wettability, and roughness, have been optimized to facilitate the rapid deposition of cell adhesion proteins. As selecting appropriate controls for bioengineering purposes remains a challenge, establishing ES scaffolds as a highly tunable platform to investigate the role of mechanical and microarchitectural properties in fibrotic disease is of great value.

## 5. Conclusions

We have established a reduction in the activation traits of NOF cultured on electrospun PCL scaffolds. Having first identified a fall in proliferation rates with more NOF occupying cell cycle phase G0, we proceeded to rule out cellular senescence as the cause. Furthermore, we identified a reduction in characteristic fibroblast activation markers, including α-sma and versican. The electrospun scaffold is stiffer than a hydrogel and enables cell spreading and cell–cell interactions; however, it is less stiff and reduces apico-basolateral polarity when compared to TCP. As such, establishing which features of the micro-fibrous PCL scaffold contribute to fibroblast regulation could grow our understanding of the matrix conditions that prevail in diseases such as fibrosis and cancer. Finally, this study represents a step toward growing fibroblasts that retain their native behaviour. This could lead to more appropriate tissue-engineered microenvironments which incorporate stromal support cells that can maintain homeostasis alongside stem cells.
